# Stercoral Ulcer Presenting in a Patient with Cauda Equina Syndrome Secondary to Postoperative Epidural Hematoma

**DOI:** 10.3390/medicina59071331

**Published:** 2023-07-19

**Authors:** Min-Seok Kang, In-Seok Son, Suk-Ha Lee, Tae-Hoon Kim

**Affiliations:** 1 Department of Orthopedic Surgery, Anam Hospital, Korea University College of Medicine, Seoul 02841, Republic of Korea; drkeith@naver.com; 2 Department of Orthopedic Surgery, Jeju National University Hospital, Jeju 63241, Republic of Korea; wiselove@naver.com; 3 Department of Orthopedic Surgery, Konkuk University Medical Center, Konkuk University School of Medicine, Seoul 05030, Republic of Korea; sthkim96@naver.com

**Keywords:** stercoral ulcer, cauda equina syndrome, postoperative, epidural hematoma

## Abstract

Chronic constipation can lead to fecal impaction in the large bowel, which can cause pressure necrosis followed by perforation, known as a stercoral ulcer. In extensive posterior thoracolumbar surgery, a long operation time, large blood loss, and perioperative narcotic use may aggravate constipation. Moreover, sacral root palsy due to cauda equina syndrome (CES) can lead to the deterioration of fecal impaction. This report describes the case of a 77-year-old woman with CES who presented with saddle anesthesia, neurogenic bladder, bowel incontinence, and paraplegia. Five days prior, she had undergone extended posterior lumbar interbody fusion from L1 to L5. Lumbar magnetic resonance imaging (MRI) showed an extended epidural hematoma. After urgent neural decompression, she gradually recovered from the saddle anesthesia, leg pain, and paraplegia over 3 weeks. Thereafter, the patient suddenly developed massive hematochezia and hemorrhagic shock. Urgent colonoscopy was performed, and a stercoral ulcer in the sigmoid colon was diagnosed. After 4 weeks of intensive care for hemorrhagic shock, pneumonia, and systemic sepsis, the patient was transferred to a general ward for intensive rehabilitation. One year after the operation, she was able to walk with assistance, and her urinary and bowel incontinence completely recovered. Chronic constipation, a common clinical problem, can sometimes cause relatively obscure but potentially life-threatening complications such as stercoral ulceration. Possible factors including advanced age, extensive spinal surgeries, prolonged operation time, significant blood loss, perioperative narcotic use, and the presence of spinal cord injury might contribute to the development of this condition. It highlights the importance of recognizing the potential development of stercoral ulcers in patients with CES and emphasizes the need for prompt diagnosis and management to avert catastrophic complications.

## 1. Introduction

Paralytic ileus is a common postoperative complication that may occur after lumbar arthrodesis surgery and can affect an individual’s quality of life during the postoperative rehabilitation period [1,2]. In the available literature, however, the risk of spontaneous bowel perforation is 3%, with an attendant mortality rate of 50% [3]. This paralytic bowel dysfunction is known to be associated with anterior retroperitoneal approach, thoracolumbar surgery, extensive surgical level (≥3-level), long operation time, higher blood loss [4], prolonged pain-related immobilization, and perioperative narcotic use. In addition, in patients with spinal cord syndrome, adynamic bowel dysfunction, weak abdominal muscles, impaired rectal sensation, and delayed colonic transit time are commonly observed [5]. A recent case report documented an occurrence of acute spontaneous perforation at the rectosigmoid junction in a patient with quadriplegia following a spinal cord injury [6]. Moreover, sacral root palsy related to cauda equina syndrome (CES) can lead to deterioration of fecal impaction [7]. Chronic adynamic bowel dysfunction can lead to fecal impaction in the large bowel, which can cause pressure necrosis followed by perforation, known as stercoral ulcers [8,9].

Stercoral ulceration is defined as an ulcer caused by pressure necrosis from hard fecal mass pressing on the colonic wall and is a relatively unknown cause of lower gastrointestinal bleeding [9]. The exact prevalence of stercoral ulceration in the general population is unknown. Stercoral ulcers area usually asymptomatic but may cause catastrophic complications [10]. However, these complications are rare in the previous literature. We report an unusual case of a patient with CES secondary to postoperative epidural hematoma who presented with hemorrhagic shock due to stercoral ulcer bleeding.

## 2. Case Presentation

**Figure 1:**
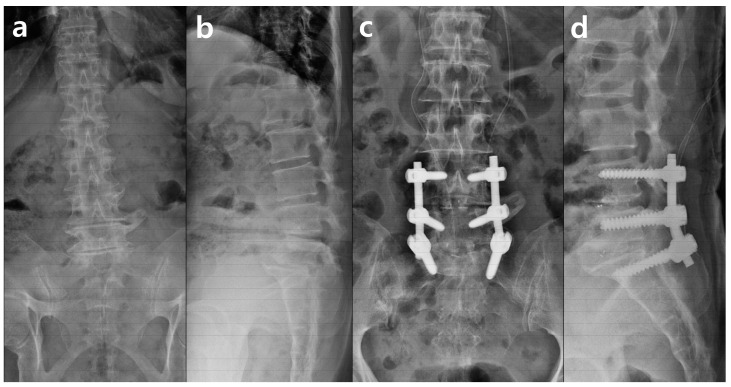
Radiographic image of X-ray at first surgery. (**a**) Preoperative lumbar AP, (**b**) preoperative lumbar lateral, (**c**) postoperative lumbar AP, and (**d**) postoperative lumbar lateral.

The patient was a 77-year-old woman who experienced severe axial back and bilateral lumbosacral radiculopathies with neurogenic claudication. The patient’s medical background included hypertension, dyslipidemia, osteoporosis, and chronic constipation. She had a history of posterior lumbar interbody fusion at L4-L5-S1 level performed a year ago for segmental kyphosis with severe central canal stenosis (Figure 1).

**Figure 2:**
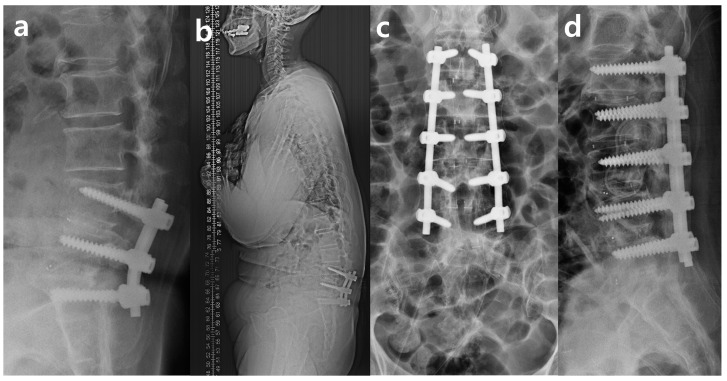
Radiographic image of X-ray at second surgery. (**a**) Preoperative lumbar lateral, (**b**) preoperative whole spine lateral, (**c**) postoperative lumbar AP, and (**d**) postoperative lumbar lateral.

However, a month after the operation, proximal junctional kyphosis occurred that gradually deteriorated, with complaints of severe gait disturbance and degenerative lumbar kyphotic deformity. The patient also suffered from chronic abdominal pain and constipation. Finally, she underwent extensive posterior lumbar instrumented fusion from L1 to L5. However, the operation was terminated with under-correction of the kyphotic deformity because of severe epidural bleeding and a prolonged operation time of more than 6 h. Immediate postoperative plain radiographs showed posterior lumbar interbody fusion from L1 to L5 and a paralytic ileus (Figure 2).

**Figure 3:**
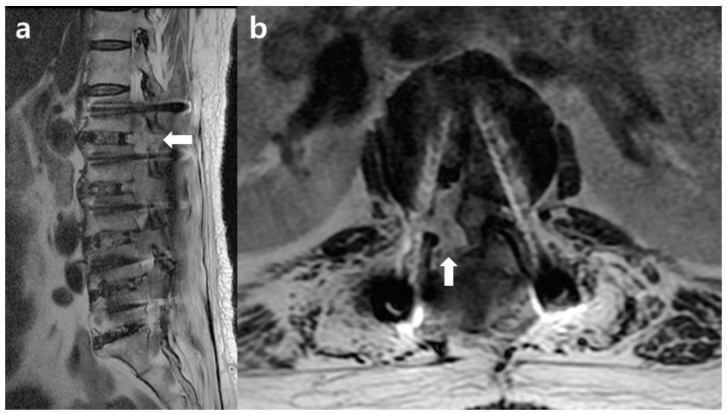
Postoperative lumbar MRI showed compressive myelopathy by extensive epidural hematoma from L1 to L3. (**a**) Sagittal; (**b**) axial cut on T2WI, MRI = magnetic resonance imaging. White arrows point to epidural hematoma.

An acceptable postoperative neurological recovery was noted, and the surgical drain was removed on the fourth postoperative day. Five days after surgery, she experienced sudden bilateral lower leg weakness, fecal incontinence, anal areflexia, and complete saddle anesthesia, and an emergency magnetic resonance imaging (MRI) study revealed CES. On MRI, compressive myelopathy due to extensive epidural hematoma from L1 to L3 was observed (Figure 3).

**Figure 4:**
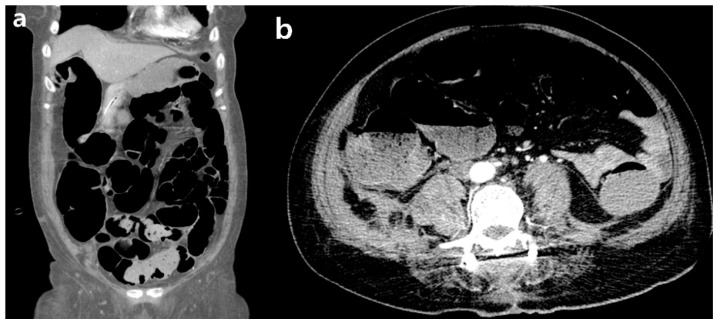
Abdominal CT angiography showed a severe small-bowel paralytic ileus. (**a**) Coronal; (**b**) axial cut.

The patient underwent urgent surgical decompression and evacuation of the epidural hematoma, and there were signs of gradual recovery from CES over the next 3 weeks. Unfortunately, in the third week after the last surgery, she presented with hypovolemic shock and severe hematochezia. To ascertain the etiology of hypovolemic shock and lower gastrointestinal bleeding, our initial approach involved conducting abdominal CT angiography. The findings from the abdominal CT angiography did not indicate any evidence of active bleeding and bowel perforation, except for the presence of small-bowel paralytic ileus (Figure 4). However, no clinical signs and symptoms associated with fecal impaction and paralytic ileus following the last operation were observed.

**Figure 5:**
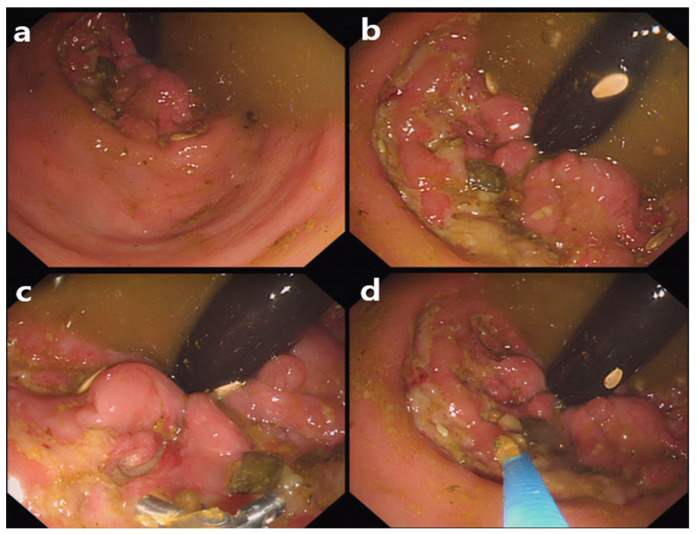
(**a**–**d**) Colonoscopy showed a circular ulcer lesion with a diameter of 1 cm at the rectosigmoid junction.

Subsequently, an emergency colonoscopy was conducted with the purpose of identifying the source of the lower gastrointestinal bleeding. On colonoscopy, a circular ulcer lesion with a diameter of 1 cm was observed at the rectosigmoid junction. This finding suggested stercoral ulcer bleeding at the rectosigmoid junction (Figure 5).

A cold biopsy was taken on the ulcer lesion located at the rectosigmoid junction, yielding a result indicative of severe chronic nonspecific inflammation accompanied by a dense infiltration of numerous eosinophils without any evidence of malignancy. She was transferred to the intensive care unit. After 4 weeks of intensive care for hemorrhagic shock, viral pneumonitis, and systemic sepsis, the patient was transferred to a general ward for intensive rehabilitation. Following an intensive rehabilitation program, the patient experienced a gradual improvement in neurological symptoms. Despite this progress, she continued to require assistance and was unable to walk independently. A follow-up colonoscopy was conducted three months later, confirming the complete healing of the ulcer lesion at the rectosigmoid junction. One year after the operation, she was able to walk with assistance, and her urinary and bowel incontinence completely resolved.

## 3. Discussion

Functional bowel disorders, including constipation, fecal impaction, and incontinence, are common gastrointestinal problems in older adults and a major source of morbidity [11]. However, these are not physiological consequences of normal aging and are often multifactorial with comorbid diseases, impaired mobility, reduced dietary fiber intake, and prescription medications contributing significantly to functional bowel disorders in many instances [12]. Lumbar spinal stenosis presents with axial back pain, lumbosacral radiculopathy, and neurogenic intermittent claudication as typical symptoms. However, lumbosacral plexopathy symptoms such as saddle hypoesthesia with bowel and bladder dysfunction may often occur while compressing the cauda equina [13]. In addition, it has been reported that the rate of adynamic bowel disorders, such as constipation and paralytic ileus, is high after thoracolumbar instrumented fusion surgery. It is associated with the anterior retroperitoneal approach, extensive surgery level, longer operation time, higher blood loss [4,14], prolonged pain-related immobilization, and perioperative narcotic use [4]. However, this gastrointestinal problem is easily overlooked in the clinical field [14]. It was reported that the risk of natural perforation secondary to paralytic enteritis is 3%, and the mortality rate is 50% [3]. Additionally, recent articles in the literature have reported that ischemia at the rectosigmoid junction is precipitated by multiple factors and might be a possible reason for the spontaneous perforation [6].

In this case of CES, a 77-year-old female patient presented with saddle anesthesia, neurogenic bladder, bowel incontinence, and paraplegia. Five days prior, the patient underwent extended posterior lumbar interbody fusion from L1 to L5 and showed acceptable progress. After urgent radiologic evaluation and proper surgical decompression, CES caused by extensive epidural hematoma showed gradual recovery over 3 weeks; however, severe hematochezia and hypovolemic shock resulted in life-threatening conditions. In patients with chronic constipation, extensive thoracolumbar surgery, longer operation time, higher blood loss, and perioperative narcotic use may have a negative effect on paralytic ileus. In fact, we prescribed 37.5 mg of tramadol for about 4 weeks for the patient’s perioperative pain control. Although the pain medication prescribed during the perioperative period of spine surgery can affect the mobility of the bowel, it is difficult to establish a direct causal relationship with stercoral ulcer. In CES, the sacral neural outflow is damaged, resulting in a lower motor nerve lesion and disruption of the reflex pathways between the spinal cord and the sigmoid–rectal wall, which becomes flaccid and hard to stimulate [5]. That is, sacral root palsy due to CES can lead to the deterioration of the fecal impaction [7]. These chronic adynamic bowel dysfunctions can lead to fecal impaction in the large bowel, which is presumed to have caused pressure necrosis followed by perforation, which is known as stercoral ulcer [8,9]. 

Fecal impaction can lead to rare but life-threatening complications such as stercoral ulcers, which are pressure ulcers caused by fecaloma [15]. Stercoral ulcers are typically circular, approximately 1 cm in diameter, and located on the antimesenteric border of the bowel. These findings can be confirmed by colonoscopy. It is a relatively unknown cause of lower gastrointestinal bleeding. The number of stercoral ulceration presenting as hematochezia is even lower [9]. Stercoral ulcers are usually asymptomatic but may cause two dreaded complications: perforation and bleeding [10]. The management of stercoral ulceration depends upon the presenting symptoms. Complications arising from stercoral ulceration may lead to 50% mortality in the population at risk due to accompanying comorbidities [9]. Surgical intervention is usually the preferred approach for stercoral ulcer perforation, while individuals experiencing severe hemorrhage may necessitate emergency resection or endoscopic therapy [10]. If peritonitis is recognized, an emergency laparotomy should be performed. Successful management depends on early diagnosis, considering the mortality rate of up to 34% [15]. This report describes a case of stercoral ulcer in a patient with CES secondary to delayed epidural hematoma after extensive posterior lumbar interbody fusion, regardless of recovery from neurological symptoms. The patient was resuscitated in an urgent and proper manner using endoscopic therapy, but long-term intensive care was required for complications of viral pneumonitis and systemic sepsis. Therefore, it was necessary to consider the factors causing this condition and note that the site of the neurologic lesion influences the pattern of bowel dysfunction.

Based on a comprehensive assessment of the paralytic ileus and fecal impaction observed on CT, the circular shape of the ulcer lesion observed during endoscopy, the location of the ulcer at the rectosigmoid junction, and the biopsy results, a final diagnosis of stercoral ulcer was established. Determining the precise pathophysiology of this patient poses challenges. However, it is highly probable that a fecaloma is most likely to form in the distal colon where the stools are in the most dehydrated form. The fecaloma is frequently impacted at the site of the rectosigmoid junction, which is also the narrowest part of the colon. Consequently, the intraluminal pressure often exceeds the capillary perfusion pressure of the bowel wall, predisposing the mucosa to necrosis and ulceration. Furthermore, it is believed that this patient developed stercoral ulceration because of several factors, encompassing underlying diseases and impaired blood circulation.

Unfortunately, it has been reported that prophylactic medications such as neostigmine and laxatives to prevent symptomatic paralytic ileus and constipation after spine surgery are ineffective [14]. Although the incidence of stercoral ulcer is very rare, it appears suddenly; therefore, it is necessary to identify and carefully observe patients who have risk factors of chronic constipation, use of narcotics and antidepressants, hypothyroidism, diabetic polyneuropathy, and hemi- or paraplegia to avoid severe complications. In addition, surgical techniques that are less invasive and cause less postoperative pain and early rehabilitation should be adopted when performing spine surgery.

We describe an unusual case of stercoral ulceration in a patient with CES secondary to postoperative epidural hematoma. Chronic constipation, a common clinical problem, can sometimes cause relatively obscure but potentially life-threatening complications such as stercoral ulceration. Possible factors, including advanced age, extensive spinal surgeries, prolonged operation time, significant blood loss, perioperative narcotic use, and the presence of spinal cord injury, might contribute to the development of this condition. This paper highlights the importance of recognizing the potential development of stercoral ulcers in patients with CES and emphasizes the need for prompt diagnosis and management to avert catastrophic complications. Further research is warranted to elucidate the underlying mechanisms and establish effective preventive strategies in this distinctive clinical scenario.

## Data Availability

Data are contained within the article.
